# A twelve‐week, four‐arm, randomized, double‐blind, placebo‐controlled, phase 2 prospective clinical trial to evaluate the efficacy and safety of an anthroposophic multimodal treatment on chronic pain in outpatients with postpolio syndrome

**DOI:** 10.1002/brb3.1590

**Published:** 2020-03-11

**Authors:** Ricardo Ghelman, Ivete Y. Akiyama, Valeria T. de Souza, Jussara Falcão, Vera Orgolini, Jorge K. Hosomi, Abrahão A. J. Quadros, Acary S. B. Oliveira

**Affiliations:** ^1^ Department of Neurology Universidade Federal de Sao Paulo São Paulo Brazil; ^2^ Department of Pediatrics Universidade de São Paulo São Paulo Brazil; ^3^ Department of Obstetrics Universidade Federal de Sao Paulo São Paulo Brazil

**Keywords:** anthroposophic medicine, motor system, neurodegenerative diseases, pain, postpolio syndrome

## Abstract

**Introduction:**

Chronic pain and fatigue are the main symptoms of postpoliomyelitis syndrome (PPS). This study aimed to evaluate the efficacy and safety of an anthroposophic multimodal treatment for chronic pain in PPS outpatients.

**Methods:**

A twelve‐week, four‐arm, randomized, double‐blind, placebo‐controlled, phase 2 prospective clinical trial was designed to compare four groups (*n* = 48): groups A and B received daily active experimental transdermal gel (ETG) or placebo gel (PTG), respectively; groups C and D received weekly external therapies, art therapies, and neurofunctional reorganization, plus either daily ETG or PTG, respectively. The pain symptoms were evaluated through a visual analogue scale (VAS), the McGill questionnaire, and thermography. Quality of life and resilience were evaluated by the WHOQOL‐BREF and Antonovsky sense of coherence questionnaires applied at baseline and after the interventions.

**Results:**

No related adverse events occurred, and 10% of the patients reports dysphagia improvement. In the groups C and D, pain reduction was statistically significant in both the placebo group (*p* = .02, *d* = 1.315) and in the ETG (*p* = .005, *d* = 2.035). However, following the week‐to‐week evolution of pain with the concomitant use of the ETG, this significant pain reduction occurred earlier from the 4th week and continued to decrease (*p* = .016, *d* = 1.369). In the group that received the complete multimodal treatment, the greatest significant benefit in increasing quality of life occurred in the physical domain and elevation in resilience with an emphasis on meaning and comprehension domains.

**Conclusions:**

The anthroposophic multimodal treatment group presented both safety and efficacy as an analgesic in the groups that received the nonpharmacological therapies, much earlier when associated with the ETG. The multimodal approach corresponded to the pattern of better efficacy for both pain reduction and improvement in quality of life and resilience.

## Graphical abstract

This study aimed to evaluate the efficacy and safety of an anthroposophic multimodal treatment for muscle and joint chronic pain in outpatients with PPS. This twelve‐week, four‐arm, parallel, randomized, double‐blind, placebo‐controlled clinical trial which were applied to evaluate pain, quality of life and resilience at baseline and after the interventions. The treatment showed increased safety and efficacy for pain and improvement of the quality of life and resilience specially in the groups that received the nonpharmacological interventions, earlier when associated to transdermal gel.

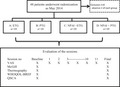

## 1 INTRODUCTION

Pain is one of the leading reasons why people turn to complementary and integrative health approaches, which may help manage pain and other symptoms that are not consistently addressed by prescription drugs and other conventional treatments (Tompkins, Hobelmann, & Compton, 2017). The use of complementary interventions in individuals with chronic pain is increasingly common worldwide, with estimates of their use ranging between 18% and 94% in the US (Breuer, 2010). For individuals with lower back pain, osteoarthritis, and fibromyalgia syndrome, there is a moderate evidence base for the use of acupuncture, meditation, chiropractic manipulation, massage therapy, and yoga (Barbour, 2000; Bronfort, Haas, Evans, Kawchuk, & Dagenais, 2008; Chung, Xu, Eken, & He, 2009; Ernst, 2006; Ernst & Posadzki, 2011; Fouladbakhsh, 2012; Kwon, Pittler, & Ernst, 2006; Manheimer, White, Berman, Forys, & Ernst, 2005; Terry, Perry, & Ernst, 2012). For this reason, the research priorities of the National Center for Complementary and Integrative Health of the National Institute of Health in the US include the study of complementary approaches to determine their safety and effectiveness for treating symptoms such as pain (Institute of Medicine (US) Committee on Advancing Pain Research, Care, and Education, 2011).

Anthroposophic medicine (AM) is a whole medical system that is widely used in Europe; it combines integrative approaches and conventional interventions with a number of specific therapeutic treatments involving a wide multiprofessional team, as well natural products (Kienle, Albonico, Baars, & Hamre, 2013; Ministério da Saúde, 2006). The review of the pain literature in the Swiss Health Technology Assessment Report on AM (2006) detected 18 studies from 189 clinical trials and systematic reviews from a wide range of diseases (Albonico, Kiene, & Kienle, 2006). The pain studies were conducted between 1973 and 2005 and evaluated 2,308 patients. In general, all of the studies showed favorable results, a high level of safety and good cost‐effectiveness ratios (Hamre et al., 2007, 2010). Vieira et al. (2012) reported an experience with 204 patients in a public outpatient center in Brazil with anthroposophical multimodal treatment for chronic pain by osteoarticular and rheumatic disorders. According to the author, 33.5% of the therapies employed were anthroposophic external therapies (AET) as compresses, bandages, medicinal baths, and clay therapy. From the specific group of 74 patients applying clay therapy, the author reported an improvement in pain management and a decrease in analgesic and anti‐inflammatory use in 75% of the patients (Vieira et al., 2012). The AET are conducted usually by anthroposophic nursing specialists that apply skills, processes, and attitudes that belong to anthroposophic knowledge of biographic development throughout the various phases of life in which care is needed (e.g., childhood, disease, disabilities, advanced age), that can promote coping and resilience in the patients.

The many survivors of the polio epidemics that occurred worldwide in the mid‐20th century constitute a population of 20 million people at risk of presenting postpolio syndrome (PPS). Since the Global Polio Eradication Initiative was launched in 1988 by the WHO, the number of cases has fallen by over 99%; however, an endemic transmission is continuing in Afghanistan, Nigeria, and Pakistan. The prevalence of PPS among people with polio sequelae has been estimated to be between 22% and 80% (Bosch, 2004). When comparing cohort studies, the prevalence of PPS was 77.2, 68, and 60% for Brazilian, North American, and Swiss population, respectively (Agre et al., 1995; Conde et al., 2009).

PPS is characterized by new muscle weakness and/or skeletal muscle fatigue that is persistent for at least 1 year, that is unrelated to other causes, and that occurs at least 15 years after a period of functional stability following the previous acute paralytic poliomyelitis illness. The diagnosis is based on a proper clinical workup in which all other possible explanations for the newly presented symptoms are ruled out (Dalakas, 1995; Dalakas et al., 1986).

Aside from new muscular fatigue, the main symptom is myalgia and joint pain, which are both progressive characteristics of the disease. Other manifestations include cold intolerance, dysphagia and sleep and respiratory disorder, dysphonia, musculoskeletal deformities, psychosocial problems, and restless legs syndrome (Lo & Robinson, 2018; Oluwasanmi et al., 2019).

The clinical and epidemiological aspects of PPS in the Brazilian population were evaluated through two studies performed in 2009 and 2012 (Conde et al., 2009; Quadros et al., 2012). The two studies included 132 and 129 patients with a mean age of 39 years after the onset of the syndrome and after a period of 30–35 years of functional stability. The most common symptoms were fatigue (87.1% and 77.5%), muscle pain (82.4% and 76.0%), and joint pain (72.0% and 79.8%), respectively. Approximately 50% of the cases were severe cases, which were defined as cases with patients who required assistance in walking and/or presented with severe sleep apnea (Quadros et al., 2012).

The pathophysiological framework relating to the genesis of PPS is based on the theory of *super training*, considering that shortly after an episode of acute poliomyelitis, the remaining motor neurons increase the number of sprouts for the reinnervation of muscle fibers after they have been denervated. About two to three decades after the acute episode, there is a tendency to overload this system, that can be accelerated depending on individual factors especially the activities and exercises carried out that promote overuse of the affected muscles. In this case of overuse and *super training*, an intense metabolic demand in the residual motor units occurs, which then triggers a process of secondary neuronal death (Orsini et al., 2015), and active inflammatory process is present in the spinal cord with increased level of cytokines in the cerebrospinal fluid but without any convincing evidence of viral reactivation (Fiorini et al., 2007).

Another hypothesis for the genesis of PPS is that it results from an autoimmune disorder, a theory that is reinforced by the presence of anti‐neurofilament antibodies in the cerebrospinal fluid (Drory et al., 1998).

An effective treatment of chronic pain in PPS requires a biopsychosocial and multidisciplinary approach including nonpharmacological strategies such as physical training for neuromusculoskeletal disorders, preventing disuse and overuse, psychological group support, voice therapy, occupational interventions associated with pharmacological drugs to managing PPS as analgesics, selective serotonin reuptake inhibitors (SSRIs) to sleep disorders, and single high dose of immunoglobulin to reduced proinflammatory cytokines present in the cerebrospinal fluid of the spinal cord. Although immunomodulation with intravenous immunoglobulins showed positive results, the benefit was modest and transient. In the case of hypoventilation, assisted ventilation would be required (Pierini & Stuifbergen, 2010; World Health Organization, 2004). Strategies should include increasing resilience as an important buffering factor for the overall wellbeing of a patient, a positive component of health, and multidimensional construct including physical, social, intellectual, emotional(mental), and spiritual level, that emphasize the balance point between an individual's resource pool and the challenges faced (Dodge, Daly, Huyton, & Sanders, 2012; Matyja, 2012; Nolvi, Brogårdh, Jacobsson, & Lexell, 2018).

The use of transcutaneous electric nerve stimulation (TENS) and acupuncture may also contribute to effective treatment to strengthen weakened muscles as well as to decrease pain (Gevirtz, 2006; Shi, 1996). In a controlled clinical trial, the use of a long‐term infrared emitter from bioceramic tissue over 4 weeks showed a significant reduction in pain intensity (8.3 ± 2.5 vs. 3.8 ± 2.5) and an improvement in sleep quality measured by polysomnographic examination that demonstrated a significant decrease in the PLM index (11 ± 14 vs. 3.7 ± 6.3) in 12 patient with cold intolerance and restless legs syndrome (Silva et al., 2009). A case report showed a positive result of *Cannabis sativa* oil to control nausea and abdominal pain as somatoform autonomic function disorder of the upper gastrointestinal tract of a eldery SPP patient, becoming a possible pharmacological option for some patients with functional disorders resistant to antiemetic and pain‐modulating drugs (Bleckwenn, Weckbecker, & Voss, 2018).

Full‐body clinical thermography (telethermography) has recently been used to monitor painful syndromes such as fibromyalgia and chronic adrenal fatigue, associated with chronic inflammatory processes, verified systemic changes due to mechanical overload as well as inflammatory changes in nonmusculoskeletal territories as well as indirect signs of pain‐induced sleep disturbance (Biasi, Fioravanti, Franci, & Marcolongo, 1994; Czaplik, Dohmeier, Barbosa Pereira, & Rossaint, 2017).

## 2 MATERIALS AND METHODS

### 2.1 Populations

The study was approved by the ethics committee of the Federal University of São Paulo (Clinical Trial Registry No. 925842). We recruited 48 patients who were admitted into the Neuromuscular Disease Outpatient Clinic of the Federal University of São Paulo. The participants provided written informed consent before study entry.

Inclusion criteria for patients were as follows: adults resident in Brazil aged 20–59‐years‐old; with confirmed paralytic poliomyelitis; who fulfilled the definition of PPS; had muscle (myofascial) and/or joint pain.

Specific exclusion criteria were as follows: had other diseases that could lead to muscle weakness; neuropathic pain; the use of a wheelchair; or who received any other type of unconventional analgesic therapy. We also excluded patients who did not agree to sign the informed consent form, who failed to perform more than three intervention sessions or who were absent on the initial and/or final evaluation days.

### 2.2 Study design

A twelve‐week, four‐arm, randomized, double‐blind, placebo‐controlled, phase 2 prospective clinical trial was designed to compare the parallel intervention of four groups with pharmacological (transdermal gel) and nonpharmacological anthroposophic clinical interventions (NPAIs).

The 48 PPS patients were randomized into blocks, with randomly selected block sizes (4:8:4:4) using SAS^®^ version 9.1 (SAS^®^ Institute), that were divided into four groups. Groups A and B received a daily experimental transdermal gel (ETG) treatment, with either active ingredients or a placebo gel (PTG), respectively; groups C and D received an NPAI weekly, plus a daily ETG or PTG treatment, respectively.

### 2.3 Interventions

The pharmacological interventions consisted of a nightly application of the ETG in groups A and C or a nightly application of PTG in groups B and D, at a dose of 1 g in the painful regions for 12 weeks. Each patient received three vials containing 30 g each, with a doser set to 1 g, and each vial was delivered every 4 weeks for use. The PTG vials contained only the inert excipient soy lecithin. The ETG vials had a final concentration of 10% of the active ingredient. The potencies and concentrations that were used followed the safety standards of the German and Brazilian homeopathic and anthroposophic pharmacopoeias, which are regulated by the National Agency of Sanitary Vigilance (ANVISA). The industrialized ETG was developed and dispensed by the Weleda Laboratory of Brazil and obeyed the blinding rules. The active ingredients of the 10% ETG were *Rhus toxicodendrum* D4 (1.66%), *Arnica montana* D3 (1.66%), *Apis mellifica*/ *Atropa belladonna* D3 (0.83%/0.83%), *Mandragora officinarum* D3 (1.66%), *Aconitum napellus* D4 (1.66%), and *Hypericum perforatum* D3 (1.66%).

The four groups of patients continued to receive their usual medical care, such as analgesics, during their admission periods. After completing the study, participants were offered the choice of receiving the ETG.

NPAIs were performed for the patients in groups C and D once a week, following a 1‐hr sequence for each therapy, which lasted over 3 hr throughout 12 sessions. All of the patients started the interventions together in a single group by receiving the anthroposophic artistic therapy (AAT), after which half of the group received the Padovan method of neurofunctional reorganization therapy (PMRN) and the other half received the anthroposophic external therapy (AET), and then, the therapies were alternated between the groups. The performance of the activities was done with respect to the functional limitations of each patient in order to avoid the patients reaching a fatigue threshold and was evaluated weekly.

AAT: The technique used was watercolor painting, according to the guidelines of the Brazilian, Dutch, and Swiss Association of Art Therapists. These sensorimotor exercises were performed with the seated patients, who applied nontoxic inks on A3 paper using a No. 20 brush. The first and last two sessions were used to evaluate the condition of each participant in terms of a comparative constitutional diagnosis through the use of a free painting exercise. The therapeutic activity was directed to the color transition using two primary colors to obtain the secondary color (green, violet, and orange).

PMRN: The therapeutic activity was conducted by licensed speech therapists and was performed in two stages, including body and oral exercises of reflex‐vegetative functions. Each stage had its own sequence of exercises, which recapitulated the neuromotor development of infants. The first stage (the homolateral program) lasted for 4 weeks and included a sequence of eight body exercises, and the second stage (the homolateral and cross‐program) lasted for 8 weeks and included a sequence of 14 body exercises, which were associated with the same oral exercises of the previous stage.

AET: The program was applied by registered nurses. This program involved the performance of a sequence of three techniques, with a total duration of 15–20 min each. The therapy site was maintained in a quiet, airy environment with subdued lighting and a pleasant temperature. A restful state was stimulated after the program through a sequence of three procedures:

1. Warmwater footbath therapy: Patients, in a sitting position, maintained their feet and calves up to 5 cm below the popliteal line in a previously sanitized bucket containing water at 40°C (104°F).
2. Rhythmic massage therapy: Patients were placed in preheated beds in the decubitus position. The slip technique was applied with massage oil and different types of circular and rhythmic pattern movements. Fabrics that came in contact with the skin were used to keep the patients warm. The massage oil had a composition of extracts including *A. Montana* L. (flower), *Betula alba* (cortex), *Helianthus annuus* L. (sunflower seed), *Vitis vinifera* L. (grape seed), *Rosmarinus off*. (leaf), and *Lavender off*. (flower) that were free of dyes, flavorings, and preservatives.
3. Bandaging: Once the massage was finished, each patient, in the dorsal decubitus position, was wrapped with the dry flannel and wool sheets, with the sheets covering the body from the neck down.

### 2.4 Data analysis

1. Primary outcome: Pain symptoms and quantitative evaluations were assessed by:
  1.1 The unidimensional scale for measure pain intensity assessment: numerical rating scales of visual analogue scale (VAS) (Gould, 2001).
    The VAS of pain was applied over the 12 weeks, with completion under the supervision of a group of nurses and/or physicians from the outpatient clinic, using a straight horizontal scale with numbers, whose extremities were defined as the extreme limits of the symptom of pain oriented from the right (“no pain”—number 0) to the left (“unbearable pain”—number 10). In groups receiving therapies (C and D), the VAS was applied before the sessions and throughout the 12 weeks. In groups that did not receive therapies (A and B), the data were collected by either weekly telephone contact or in person when they arrived to pick up their gel bottles once a month.
  1.2 Questionnaire for multidimensional pain evaluation (McGill Pain Questionnaire).
    This self‐reporting measure of pain questionnaire allows for the communication of the sensorial, affective, and evaluative qualities of the painful phenomena (Melzack, 1975).
  1.3 Transcutaneous thermography using the FLIR T420 High‐performance infrared camera (thermal sensitivity < 0.035°C). Full‐body thermal images were obtained under a controlled temperature (Lahiri, Bagavathiappan, Jayakumar, & Philip, 2012).
2. Secondary outcome: Quality of life and resilience.
3. Quality of Life Questionnaires:
  3.1 WHOQOL‐BREF—Quality of life assessment questionnaire, which produces a multidimensional profile of scores between domains and subdomains (facets), in the abbreviated version of 26 items containing the physical, psychological, social relations, and environmental domains (The WHOQOL Group, 1997).
  3.2 Antonovsky's sense of coherence Questionnaire (QSCA): resilience assessment (Orientation to Life Questionnaire to measure the sense of coherence) with 29 questions. This assessment consists of questions distributed according to three components: 11 items measuring comprehensibility, 10 items measuring manageability, and eight items measuring meaningfulness (Eriksson & Lindström, 2007).

**FIGURE 1:**
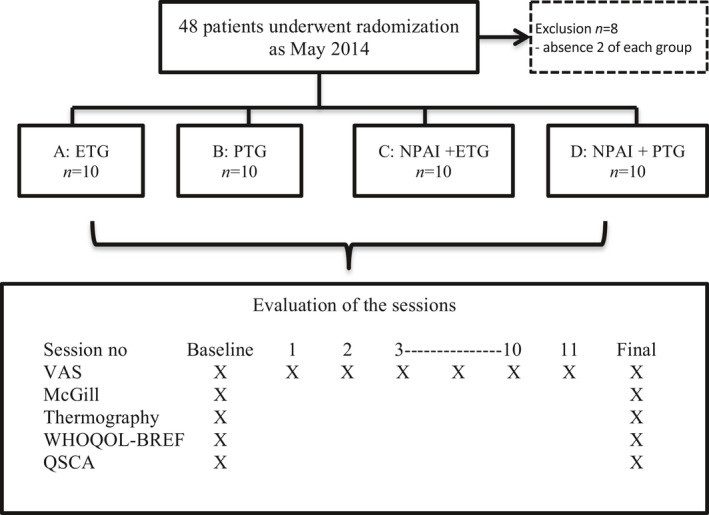
Trial diagram

The questionnaires and thermographies were applied in all groups at baseline and after the 12‐week interventions (Figure 1). A weekly safety evaluation was conducted, based on spontaneous complaints by the study subjects, with a special emphasis on cutaneous allergies.

For the statistical analysis, the Student *t* test was used for samples that were correlated with a uniform distribution profile, and Wilcoxon nonparametric paired test was used for nonuniform distributions of the data. For a correlation with severity, the Pearson correlation test was used for samples with a regular distribution profile, and a Spearman correlation test was used for simples with an irregular data distribution. The statistical analysis was performed with SAS^®^ version 9.2. It used the sample power of Minitab (power and sample size) and calculated the main statistical parameters (mean and standard deviation) of the protocol. As we analyzed eight variables (VAS, three QSCA domains, and four WHOQOL domains), we determined a power of the sample at 80% and thus calculated the nominal margin of error, considering the statistical margin of error of 5%, that is, confidence interval of 95%. The effect size was classified following Cohen parameters into five categories: very small (*d* < 0.2), small (*d* = 0.2), medium (*d* = 0.5), large (*d* = 0.8), and very large (*d* > 1.2) (Cohen, 1988).

## 3 RESULTS

Of the 48 recruited patients, we excluded eight patients due to the excessive absence of six patients (one in the ETG group, two in the PTG group, two in the ETG + NPAI group, and one in the PTG + NPAI group) and the lack of attendance for the final evaluation in two patients (one in the PTG + NPAI group and one in the ETG group) in two of the groups. Thus, the sample size for the statistical analyses was 40 patients.

Considering the statistical analysis of these 40 patients and the sample power at 0.8, we found the value of the nominal difference margin of error for each of the nine variables (VAS, four QSCA domains, and four WHOQOL domains). The nominal differences of QSCA domains ranged between 3.88 and 9.47 and WHOQOL domains between 5.88 and 8.9, and 1.34 in VAS value, all acceptable for statistical analysis.

The assessment of adverse events was actively conducted by the nursing and medical staff during the weekly assessment as a specific issue concomitant with the assessment of VAS and at the end of the protocol in a comprehensive manner, in addition to the guidance of spontaneous adverse event reporting throughout the 12‐week intervention and after treatment. No study‐related adverse events occurred. There were no reports of cutaneous allergies induced by the application of the transdermal gel or by the application of arnica oil during external therapies.

We observed reports of dysphagia improvement in four patients who received therapies (three in the ETG + NPAI group and one in the PTG + NPAI group), which represented 10% of the total sample of 40 patients. We postulated that the PMRN intervention should have contributed more directly to this improvement, as a result of the oral exercises.

The thermographic examinations showed a pattern of alterations that was observed in almost all of the patients. These alterations were maintained before and after the interventions and practically characterized the thermal signature of PPS.

The main findings were a pattern of thermal asymmetry between the upper and lower regions of the body, which showed significant hypothermia in the affected limb and hyperthermal signals of osteoarthritis and sacroiliitis of the hip. In addition to these mechanical components that were linked to an imbalance of the musculoskeletal structure, we found systemic signs such as hyper‐radiation in palpebral region due to venous congestion, hyper‐radiation in the abdominal area, and also in the cervicothoracic regions including interscapular area of trapezius, rhomboid, and supraclavicular muscles, which characterized the signal of mantle.

These systemic findings are due to nonrepairing sleep and fatigue expressed in the periocular region, intestinal dysbiosis as a sign of gastrointestinal dysfunction, and exacerbated thermogenesis in brown fat tissue (BAT), mediated by adrenergic activity induced by cold temperatures and/or stress as signal of mantle (Biasi et al., 1994; Costa, Maia, Brioschi, & Machado, 2017; Larson, Pardo, & Pasley, 2014).

### 3.1 Pain outcome

The analyses showed a significant reduction in pain in the groups that employed therapies (NPAI), both in the groups accompanied with placebo (VAS *p* = .021) and in the ETG group (*p* = .017), according to Table 1.

**TABLE 1:** Pain (VAS) in the four groups before and after the 12‐week intervention

Groups	Mean	Median	*SD*	Q1	Q3	*N*	CI	*p*‐value
A‐ETG								
Before	6.29	6.42	1.93	4.27	7.71	11	1.14	.075
After	4.67	5.00	2.89	2.65	6.00	11	1.71	
B‐PTG								
Before	5.52	5.86	1.21	4.96	6.11	6	0.97	.116
After	4.10	3.06	2.66	2.31	6.05	6	2.13	
C‐ETG + NPAI								
Before	5.44	5.00	1.34	4.73	6.36	8	0.93	.017*
After	2.19	1.40	2.18	0.92	2.75	8	1.51	
D‐PTG + NPAI								
Before	5.43	5.67	2.06	4.00	6.14	9	1.34	.021*
After	2.47	2.20	1.80	1.00	4.00	9	1.17	

Abbreviations: CI, confidence interval; ETG, experimental transdermal gel with active ingredients; NPAI, nonpharmacological anthroposophic clinical intervention; PTG, placebo transdermal gel; *SD*, standard deviation; VAS, visual analogue scale.

* Statistically significant *p*‐value < .05.

In the groups without therapies, there was a reduction in the amount of pain of approximately 20.7% in the ETG group (*p* = .268) and approximately 24.8% in the PTG group (*p* = .317), but these reductions were not statistically significant, as shown in Table 1. Such findings are consistent with the placebo response rates that are frequently much higher than the often‐cited one‐third response rate (Turner, Deyo, Loeser, Von Korff, & Fordyce, 1994).

In the groups receiving NPAI, as shown in Table 2, pain reduction was statistically significant with very large Cohen's *d* effect size in both the placebo group (51.1%, *p* = .02, *d* = 1.315) and in the ETG (63.4%, *p* = .005, *d* = 2.035), thus demonstrating an independent analgesic effect of NPAI.

**TABLE 2:** Evolution of pain (VAS) in each weekly session in the four groups

	Mean	Median	*SD*	Q1	Q3	*N*	CI	*p*‐value	*d*
A‐ETG									
Baseline	5.89	6.15	2.32	4.19	7.60	12	1.31	—	—
1ª session	5.28	5.38	2.09	3.93	6.79	12	1.18	.435	—
2ª session	5.88	6.58	2.54	4.27	7.43	12	1.44	.908	—
3ª session	4.96	5.17	2.59	3.90	7.53	12	1.46	.340	—
4ª session	5.18	5.29	2.53	3.90	6.67	11	1.49	.442	—
5ª session	5.34	6.10	2.82	3.39	7.60	10	1.75	.792	—
6ª session	4.24	4.33	3.05	1.99	6.88	10	1.89	.187	—
7ª session	5.52	5.80	3.02	2.50	8.11	11	1.78	.806	—
8ª session	4.75	5.08	3.00	2.30	7.29	10	1.86	.429	—
9ª session	5.27	4.39	2.44	4.04	7.29	10	1.51	.553	—
10ª session	4.73	4.54	2.82	2.33	5.58	9	1.84	.320	—
11ª session	4.68	4.60	3.23	1.78	6.24	8	2.24	.440	—
Final	4.67	5.00	2.89	2.65	6.00	11	1.71	.268	—
B‐ETG									
Baseline	5.45	5.71	1.12	4.86	6.07	7	0.83	—	—
1ª session	4.74	5.29	1.78	3.81	5.45	7	1.32	.406	—
2ª session	4.75	5.71	2.28	2.69	6.23	7	1.69	.608	—
3ª session	4.89	5.75	2.45	2.88	6.79	7	1.82	.798	—
4ª session	4.63	4.67	2.61	2.64	6.39	7	1.94	.406	—
5ª session	4.70	3.94	2.71	2.38	6.91	6	2.17	.568	—
6ª session	4.25	3.57	2.50	2.39	6.22	6	2.00	.352	—
7ª session	5.17	3.89	2.14	3.53	7.08	7	1.58	.749	—
8ª session	5.11	4.88	2.66	3.16	7.21	7	1.97	.609	—
9ª session	4.45	3.30	2.44	2.81	6.21	7	1.81	.277	—
10ª session	4.84	4.46	2.78	2.98	7.21	6	2.22	.568	—
11ª session	4.41	4.00	2.51	2.43	6.24	7	1.86	.406	—
Final	4.10	3.06	2.66	2.31	6.05	6	2.13	.317	—
C‐NPAI + ETG									
Baseline	5.44	5.00	1.34	4.73	6.36	8	0.93	—	—
1ª session	4.58	4.40	1.86	3.00	5.90	10	1.15	.265	0.552
2ª session	4.04	3.73	2.19	2.74	4.59	10	1.36	.075	0.796
3ª session	4.17	3.60	1.76	2.67	5.29	9	1.15	.149	0.857
4ª session	2.92	2.37	2.32	1.29	4.07	10	1.44	.016*	1.369
5ª session	3.86	2.95	3.02	1.75	5.36	7	2.23	.132	0.746
6ª session	2.42	2.20	1.36	1.45	3.49	6	1.09	.004*	2.419
7ª session	2.12	1.49	1.71	0.99	2.63	8	1.19	.006*	2.310
8ª session	2.97	2.40	2.48	1.35	4.51	7	1.84	.064	1.360
9ª session	1.80	0.88	2.38	0.40	1.20	9	1.56	.009*	1.973
10ª session	1.90	0.80	2.36	0.50	1.57	9	1.54	.012*	1.931
11ª session	2.43	1.80	2.20	0.78	3.55	9	1.44	.016*	1.733
Final	1.99	1.17	2.13	0.38	2.20	9	1.39	.005*	2.035
D‐NPAI + PTG									
Baseline	5.05	5.18	2.29	3.38	6.13	10	1.42	—	—
1ª session	4.24	4.41	2.00	3.92	4.81	10	1.24	.427	0.397
2ª session	4.43	3.93	2.35	3.25	5.23	8	1.63	.477	0.284
3ª session	3.12	3.60	1.52	2.33	3.67	9	0.99	.060	1.038
4ª session	3.00	2.71	2.20	1.67	4.44	9	1.44	.102	0.964
5ª session	4.28	4.22	2.07	2.68	6.11	6	1.66	.664	0.372
6ª session	3.15	2.78	2.27	1.79	4.42	6	1.82	.193	0.890
7ª session	3.60	4.27	2.84	1.50	4.98	7	2.11	.242	0.611
8ª session	3.80	3.92	2.02	2.92	5.01	8	1.40	.374	0.609
9ª session	3.09	2.89	2.11	2.00	4.17	7	1.57	.143	0.940
10ª session	2.74	2.48	2.16	1.25	3.69	8	1.49	.051	1.097
11ª session	3.12	3.64	2.42	1.00	4.33	9	1.58	.086	0.867
Final	2.47	2.20	1.80	1.00	4.00	9	1.17	.020*	1.315

Abbreviations: CI, confidence interval; d, Cohen's d effect size; ETG, experimental transdermal gel with active ingredients; NPAI, nonpharmacological anthroposophic clinical intervention; PTG, placebo transdermal gel; *SD*, standard deviation; VAS, visual analogue scale.

* Statistically significant *p*‐value < .05.

**FIGURE 2:**
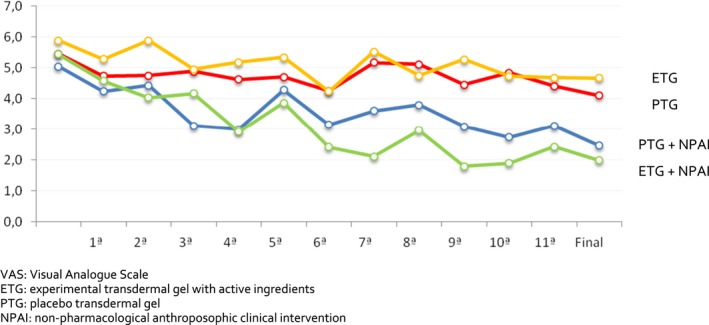
Pain evolution by VAS in the four groups during the 12 weeks of intervention

However, following the week‐to‐week quantitative evolution of pain in the groups that received therapies (NPAI), we noticed that, with the concomitant use of the ETG, this significant pain reduction with very large effect size occurred earlier and always continued to decrease from the 4th week (VAS mean of 2.92, decreased from the initial VAS mean of 5.44, *p* = .016, *d* = 1.369), while in the placebo group, the significant decrease occurred only at week 12 (Table 2; Figure 2).

A multidimensional pain assessment by the McGill questionnaire showed maintenance of the qualitative pain pattern, both before and after the 12‐week interventions.

### 3.2 Quality of life outcome

In the groups that received the NPAI with the addition of the ETG, there was significant improvement of quality of life with very large effect size, which was evaluated by the WHOQOL‐BREF questionnaire in the environmental (*p* = .016, *d* = 1.532) and physical (*p* = .007, *d* = 1.594) domains and almost large effect size in social (*p* = .043, *d* = 0.766) domain, compared to the placebo group (Table 3).

**TABLE 3:** Quality of life by Gel action in various WHOQOL‐BREF domains

	Mean	Median	*SD*	Q1	Q3	*N*	CI	*p*‐value	*d*
ENVIR									
NPAI									
PTG	−3.57	−3.13	6.85	−7.82	0.00	7	5.08	.016*	1.532
ETG	8.20	9.37	9.29	2.34	15.63	8	6.44		
No NPAI									
PTG	−3.12	−6.25	6.25	−6.25	0.00	5	5.48	.784	0.632
ETG	3.12	0.00	12.30	−3.13	6.25	9	8.04		
Both									
PTG	−2.23	−1.57	6.54	−6.25	2.35	14	3.42	.075	0.787
ETG	4.77	3.12	10.69	−3.13	15.63	19	4.81		
PHYS									
NPAI									
PTG	1.53	3.57	9.42	−3.57	8.93	7	6.98	.007*	1.594
ETG	19.64	17.86	14.16	11.60	32.14	8	9.81		
No NPAI									
PTG	0.00	3.57	5.05	−3.57	3.57	5	4.43	.640	0.635
ETG	3.97	3.57	7.46	0.00	7.14	9	4.87		
Both									
PTG	2.04	3.57	8.37	−2.68	6.25	14	4.39	.043*	0.757
ETG	10.34	3.57	13.03	1.79	16.07	19	5.86		
SOCIAL									
NPAI									
PTG	−0.60	0.00	7.77	−4.17	4.17	7	5.75	.043*	0.766
ETG	10.42	16.67	19.79	6.25	25.00	8	13.72		
No NPAI									
PTG	6.67	0.00	9.13	0.00	16.67	5	8.00	.649	0.109
ETG	9.26	8.33	30.75	−8.33	25.00	9	20.09		
Both									
PTG	2.08	0.00	8.76	0.00	8.33	14	4.59	.330	0.227
ETG	6.58	8.33	25.85	−12.50	25.00	19	11.62		
ETG + NPAI									
PHYS									
Before	26.98	28.6	13.26	17.9	32.1	9	8.66	.012*	1.550
After	47.62	57.1	14.94	32.1	60.7	9	9.76		
PSYCH									
Before	47.92	47.9	16.58	34.4	63.5	10	10.28	0.028*	0.508
After	55.83	56.3	16.22	51.0	64.6	10	10.05		
SOCIAL									
Before	45.00	41.7	19.33	33.3	58.3	10	11.98	0.153	0.505
After	54.17	54.2	18.94	50.0	70.8	10	11.74		
ENVIR									
Before	45.14	40.6	13.45	40.6	53.1	9	8.79	0.035*	0.683
After	54.17	56.3	14.57	40.6	59.4	9	9.52		

Abbreviations: CI, confidence interval; *d*, Cohen's d effect size; ETG, experimental transdermal gel with active ingredients; NPAI, nonpharmacological anthroposophic clinical intervention; PTG, placebo transdermal gel; *SD*, standard deviation; WHOQOL‐BREF, WHO Quality of life assessment questionnaire abbreviated version.

* Statistically significant *p*‐value < .05.

In the group that received the complete treatment (ETG + NPAI), which represented the multimodal model, the greatest significant benefit in increasing quality of life occurred in the physical domain (*p* = .012, *d* = 1.55) with very large effect size, and secondarily in the psychological (*p* = .028, *d* = 0.508) and environmental (*p* = .035, *d* = 0.683) domains with medium effect size as shown in Table 3.

Resilience, which was measured by QSCA, was significantly elevated with the use of the transdermal gel, showing a medium effect size (*p* = .008, *d* = 0.671). Nevertheless, when the gel was associated with the therapies (NPAI), there was a large effect size (*p* = .028, *d* = 0.972) as shown in Table 4.

**TABLE 4:** Resilience by Gel action in the QSCA domains

	Mean	Median	*SD*	Q1	Q3	*N*	CI	*p*‐value	*d*
PTG without NPAI									
Comprehension									
Before	43.55	44	11.00	36	51	11	6.50	0.066	0.554
After	49.27	49	10.67	43	53	11	6.30		
Meaning									
Before	38.45	36	7.50	33	45	11	4.43	0.539	0.114
After	39.36	41	9.17	34	44	11	5.42		
Manageability									
Before	45.36	46	5.87	40	49	11	3.47	0.373	0.285
After	47.27	45	8.04	42	50	11	4.75		
Total									
Before	124.73	119	20.71	108	138	11	12.24	0.008*	0.671
After	139.55	131	25.36	126	154	11	14.99		
PTG + NPAI									
Comprehension									
Before	39.00	39	10.91	36	46	9	7.13	0.030*	0.996
After	47.56	46	6.86	44	51	9	4.48		
Meaning									
Before	34.44	36	7.54	28	41	9	4.92	0.015*	0.787
After	40.78	43	9.44	33	48	9	6.17		
Manageability									
Before	42.44	42	5.68	39	48	9	3.71	0.138	0.497
After	45.89	45	8.72	38	49	9	5.70		
Total									
Before	116.11	118	19.48	97	131	9	12.73	0.028*	0.972
After	133.89	128	19.32	125	136	9	12.62		
Meaning NPAI									
PTG									
Yes	−0.44	3.00	8.29	−8.00	5.00	9	5.42	0.560	0.249
No	−2.43	−1.00	8.89	−6.00	0.50	7	6.58		
ETG									
Yes	6.33	7.00	5.17	6.00	7.00	9	3.38	0.047*	1.083
No	0.91	1.00	5.36	−3.50	5.00	11	3.17		
Both									
Yes	2.94	5.50	7.56	−0.50	7.00	18	3.49	0.124	0.473
No	−0.39	0.00	6.90	−4.00	3.25	18	3.19		

Abbreviations: CI, confidence interval; *d*, Cohen's d effect size; ETG, experimental transdermal gel with active ingredients; NPAI, nonpharmacological anthroposophic clinical intervention; PTG, placebo transdermal gel; QSCA, Antonovsky's sense of coherence Questionnaire; *SD*, standard deviation.

* Statistically significant *p*‐value < .05.

However, when comparing the concomitant use of the ETG with the therapies versus the use of the gel alone, there was a higher degree of elevation in resilience in the full intervention (Table 4), with an emphasis on meaning (*p* = .015, *d* = 0.787) and on comprehension domains (*p* = .030, *d* = 0.996) with large effect size.

## 4 CONCLUSION

We concluded that the multimodal anthroposophic treatment presented both safety and efficacy as an analgesic in the groups that received the nonpharmacological therapies as an independent intervention, but exhibited these properties much earlier when associated with the ETG compared to the placebo gel. In the groups that used ETG, especially when associated with the therapies, there was an improvement in both the quality of life and the degree of resilience. The multimodal use of this anthroposophic treatment, which combines topical pharmacological with nonpharmacological therapy, corresponded to the pattern of better efficacy in this study for both pain reduction and improvement in quality of life and resilience. Typical thermography findings show a comorbidity with other painful, sleep disturbance, and fatigue syndromes, which should be further investigated. Although this study has a small sample, the effect size of the sample allowed us to demonstrate the benefit of this complementary approach with emphasis on pain, resilience, and quality of life. Multicenter clinical studies evaluating the medium‐ and long‐term analgesic effect of this multimodal anthroposophic treatment should be encouraged.

## CONFLICT OF INTERESTS

None declared.

## AUTHOR CONTRIBUTION

Ricardo Ghelman conceived the study, participated in its design, collected the data, planned the analysis, participated in clinical investigation, and led the drafting of the manuscript. Jorge K. Hosomi conducted the randomization and the blinding process until data analysis. Abrahão A. J. Quadros participated in the clinical investigation, especially in recruitment. Ivete Y. Akiyama participated in the clinical investigation as the leader of the physician team. Valeria Tiveron de Souza participated in the clinical investigation as the leader of the nurse team. Jussara Falcão participated in the clinical investigation as the leader of the speech therapists team. Vera Orgolini participated in the clinical investigation as the leader of the art therapist team. Acary S. B. Oliveira participated in designing the study, the clinical investigation, and the drafting of the manuscript.

## Data Availability

The data that support the findings of this study are available in Brazilian Clinical Trial Registry/National Health Council—Plataforma Brasil, reference number [925842]. These data were derived from the following resources available in the public domain: Ministry of Science, Technology and Innovation, through the National Council for Scientific and Technological Development (CNPq), Department of Science and Technology of the Ministry of Health of Brazil (Grant/ Award Reference Number: 401309/2013‐4) and National Postdoctoral Program of the Coordination of Improvement of Higher Education Personnel (CAPES), Foundation of the Ministry of Education, carried out with the Department of Neurology and Neurosurgery of the Federal University of São Paulo as Ricardo Ghelman's Postdoctoral thesis in Neuroscience, after approval by the Research Ethics Committee (Reference number. 19826).
